# The effect of regular nursing rounds on patients' comfort and satisfaction, and violence against nurses in surgical ward

**DOI:** 10.1016/j.heliyon.2023.e17708

**Published:** 2023-07-01

**Authors:** Zohre Roustaei, Narges Sadeghi, Azim Azizi, Mostafa Eghbalian, Sahar Dehdar Karsidani

**Affiliations:** a Malayer Mehr Hospital, Hamadan University of Medical Sciences, Hamadan, Iran; b Student Research Committee, School of Nursing and Midwifery, Hamadan University of Medical Sciences, Hamadan, Iran; c School of Nursing and Midwifery, Hamadan University of Medical Sciences, Hamadan, Iran; d Department of Biostatistics and Epidemiology, School of Public Health, Kerman University of Medical Sciences, Kerman, Iran; f Hamadan University of Medical Sciences, Hamadan, Iran

**Keywords:** Regular nursing rounds, Violence in the workplace, Nurse, Hospital, Patient satisfaction, Patient comfort

## Abstract

**Background:**

Patients' satisfaction and comfort are known as the quality indicators of nursing care. Nowadays, violence against nurses has an increasing trend. Regular nursing rounds are one of the caring programs that help improve these indicators. This study aimed to examine the effect of regular nursing rounds on patients' comfort, satisfaction, and violence against nurses.

**Materials and methods:**

This quasi-experimental study was conducted in two groups consisting of 100 patients and 35 nurses in a surgery ward in the northwest of Iran. The satisfaction with nursing care quality questionnaire, Kolcaba's general comfort questionnaire, and work environment violence were used for data collection. In the present study, the control and intervention groups were selected using a simple sampling method. The control group received routine care only; however, the intervention group received a regular nursing round program every 2 h from the second day of their admission for three days. The satisfaction questionnaire and comfort scale were completed on the second and fifth days of admission, and the evaluation of violence against nurses was performed from the second to the fourth day. The results were analyzed using chi-square, Fisher, independent t, and paired t tests.

**Results:**

Before the intervention, no statistically significant difference was observed between the two groups in terms of demographic and dependent variables (p > 0/05). After the intervention, statistically significant differences were observed among the mean scores of satisfaction with nursing care (p < 0/001), comfort (p < 0.001), and violence against nurses (p = 0.041) between the two study groups, so that in the intervention group, the patients’ satisfaction and comfort increased and violence against nurses reduced during the intervention period.

**Conclusions:**

The use of regular nursing rounds had a positive effect on the study results. Therefore, it is recommended that nurse managers design, implement, encourage, and evaluate regular nursing rounds to improve nursing care.

## 1 Introduction

Nowadays, quality assessment and health system improvement are emphasized. Nurses play a significant role in improving the quality of patient care due to their continuous presence at the patient's bedside. One of the important indicators for assessing the quality of health care is the patient's satisfaction with nursing care [1].

Another component of assessing the quality of service is the comfort of patients. Comfort is a fundamental human need at all stages of life and an outcome of nursing practice. Moreover, comfort is a comprehensive, subjective, dynamic, and positive experience based on the satisfaction gained from fulfilling individual needs in one or more dimensions (including physical, mental, social, and spiritual dimensions) [2].

Patients have the right to receive quality nursing care. However, the results of a study previously conducted in Iran showed that only 39.7% of patients had complete satisfaction with nursing care services [3]. Additionally, the results of Aiken et al.‘s study showed that the quality of nursing care and patient satisfaction were low in Ireland and Greece [4]. Although in studies on the improvement of patient satisfaction, proper communication and paying respect to patients have been emphasized, the impact of these programs has not been remarkable [5]. Despite great efforts, we have not yet been able to improve patients' satisfaction with nursing care [6]. Therefore, determining the extent to which nursing care promotion can affect patients' satisfaction is very important.

Providing comfort can prevent the adverse physiological outcomes caused by the disease and create a positive effect on the physical and mental status of patients [7]. Moreover, it can accelerate the rate of discharge from the hospital [8]. Feeling comfortable is the most sought-after need of patients for relief from difficult and stressful situations [9]. It seems that the provision of patient comfort has gradually become one of the least important nursing priorities, and this important role of nurses has changed over time [10]. Some nurses are unwilling to engage in the primary aspects of nursing care, like patient comfort. Through listening to patients' opinions, the development of a new nursing care system called “regular nursing rounds” seems very essential [11].

One of the results related to the reduced quality of nursing care is the increasing rate of violence against nurses. Nurses are at increased risk of violence due to having direct contact with patients and their relatives three times more likely than other healthcare professionals [12]. The evaluations of the outpatient department indicated subjects of concern to the administration. The important causes of workplace violence, patient dissatisfaction, and lack of safety in China are rejection of a request, long wait times, dissatisfaction with the treatment process, dissatisfaction with the treatment outcome, and death-related issues [13]. Accordingly, there is no practical method for its prevention.

Regular nursing rounds, or patient-comfort rounds, were first devised by Castledine et al., in 2005 with the aim of improving patient care [11]. The program included the improvement of communication, regular reviews, providing effective and timely interventions to meet patients’ needs and concerns, and evaluating the consequences of interventions on their physical and mental health statuses [1,5,6].

Regular nursing rounds described with a variety of methods provide suggestions for converting the theory of nurse-patient interaction into practice. Considerable evidence exists that it has been used in some settings to provide beneficial effects, particularly in improving service quality, patient safety, and patient satisfaction [1,14]. A study showed that regular nursing rounds could increase patients' satisfaction [15]. It has been shown that the use of clinical rounds decreases stress and uncertainty in patients [16]. However, the studies by Kalman et al. [17] and Gardner et al. [18] showed that hourly rounds have no effect on patients’ satisfaction.

Nursing services in Iran are provided as a case method. Depending on the patient's needs, the care of 2–10 patients is assigned to the nurse [19]. Ahmadi Chenari et al.'s study showed that nursing services provided to patients could not meet the demands and expectations of patients in Iran [20]. The results of Zareh et al. showed that about one-third of the patients were satisfied with the quality of the nursing services provided [21].

Therefore, considering contradictory results on the effect of regular nursing rounds on patients’ satisfaction [[15], [16], [17], [18]], the need to distinguish between patient satisfaction with nursing care and other aspects of health care [22], and also due to the increase in violence against nurses, the discomfort of patients if the quality of nursing care decreases, and reducing satisfaction with nursing services, it seems that the use of regular nursing rounds can be an effective technique to solve these problems. Therefore, this study was conducted to determine the effects of regular nursing rounds on patients' comfort and satisfaction and violence against nurses in the surgical ward.

## 2 Materials and methods

### 2.1 Study design

This semi-experimental study was done in intervention and control groups before and after in Malayer Hospital, northwest of Iran. The study was done in the general surgery ward, which has 60 active beds and 40 full-time nurses. This study was conducted for six months in 2021.

### 2.2 Participants

Nurses and patients were selected through a convenience sampling method. In order to prevent bias, first the patients of the control group were entered into the study. After completing the sampling of the control group and discharging the last person from this group from the hospital, the intervention group's patients were entered in the study.

In this study, first, 35 nurses working in the department entered the study as convenience sampling and took care of the patients of the control group. After the discharge of the last patient of the control group, these nurses received training on how to perform regular nursing rounds and took care of the patients who appeared in the role of intervention group nurses during this period.

Adult patients admitted to the general surgery ward were recruited based on the following inclusion criteria: age more than 18 years old; lack of psychological problems; ability to communicate, read, write, and understand Persian; and absence of any chronic disease affecting patients' comfort. For nurses, criterion was having at least one year work experience Moreover exclusion criteria were unwillingness cooperate every stage research being discharged hospital transferred other wards before completing intervention any acute problem affecting patient's health status besides their main health problem.

Based on the mean and standard deviation of study by Negarandeh et al. [3], σ = 2/44, δ = 3/3, α = 0.05, β = 95/01, and 10% probability of drop out, the same size was estimated to be 50 patients for each group. As well, a total of 35 employed nurses in the general surgery ward, who were directly caring patients, were entered into the study by census.

Sample size formula:

$$n=\frac{{\left(z_{1}-\alpha /2+z_{1}-\beta \right)}^{2}\sigma^{2}}{\delta^{2}}$$

### 2.3 Measurements

#### 2.3.1 Demographic information

The demographic information of the included patients consisted of gender, marital status, age, educational level, economic status, hospitalization history, and duration of hospitalization; and age, gender, and job history for nurses.

#### 2.3.2 Satisfaction with nursing care quality questionnaire (PSNCQQ)

Satisfaction with nursing care quality questionnaire was developed by Laschinger et al. [23]. This questionnaire was used and validated in previous Iranian studies. It was translated to Persian language by Negarandeh et al. in Iran [24]. The Persian version consisted of 21 items and 4 domains, including knowledge and professional skills (7 items), communication (4 items), nursing ethics (5 items), and patient education (5 items) scored on a five-point Likert scale from excellent (5 points) to very weak (1 point) and the total score was between 21 and 105 [3,24]. To prevent data collection bias, the questionnaires were provided to the patients by someone other than the research team.

#### 2.3.3 Patient's comfort questionnaire

Kolcaba's general comfort questionnaire (GCQ) was used to assess the patients' comfort, which was translated by Soltani in 2014. Accordingly, it has 21 questions on feeling comfortable in three physical, environmental, and psychological dimensions, with 6, 7, and 8 questions, respectively. Moreover, it is scored on a four-point Likert scale as Strongly Agree (score 4), Agree (score 3), disagree (score 2), and strongly disagree (score 1). In total, the answers to the questionnaire are scored as a minimum of 21 up to a maximum of 84 [25].

#### 2.3.4 Workplace violence in the health sector questionnaire

Workplace violence in the health sector questionnaire was used to investigate the incidence of violence against nurses by patients or their relatives. It has 43 items, including questions on demographic data (11 items), threat (10 items), verbal violence (10 items), and physical violence (12 items) [26]. Reliability of this questionnaire was evaluated by Imani et al. using a test-retest method (r = 0.8) [26]. In this study, the reliability of the instrument was assessed by 15 nurses within a week-interval using a test-retest method. The correlation coefficient was reported as r = 0.81.

### 2.4 Validity and reliability

In the Negarande et al.‘s study, validity of PSNCQQ has been confirmed, the mean of CVI and CVR was 0.85 and 0.77 respectively and indicated a good content validity for this questionnaire [24] and the questionnaire reliability was also reported by Cronbach's alpha as 0.82 [24], In addition, in the present study, Cronbach's alpha of the questionnaire was estimated at 0.87 and its dimensions were between 0.81 and 0.92. In the Soltani's study, the CVR and CVI values for the GCQ were obtained as 0.89 and 0.85, respectively [27]. Also, the Cronbach's alpha value was obtained as 0.88 for it [25]. In the present study, the Cronbach's alpha value for the GCQ was calculated as 0.85. Validity of workplace violence in the health sector questionnaire was evaluated by Noorullahi et al. In their study content validity index (CVI) was obtained more than 0.79 and content validity ratio (CVR) was more than 0.70 for all questions. Also, Cronbach's alpha coefficient with 0.89 confirmed the reliability [28]. In this study, the reliability of the instrument was assessed by 15 nurses within a week-interval using a test-retest method. The correlation coefficient was reported as r = 0.81.

### 2.5 Data collection

The patients completed satisfaction and comfort questionnaires as a self-report on the hospitalization second day morning. The questionnaires for illiterate participants was completed by interview.

#### 2.5.1 Control group

In the control group, the patients received routine care from the second to the fourth day of their hospitalized for three days by nursing ward who participated in this study.

#### 2.5.2 Intervention group

After discharging last patient control group researcher held three teaching sessions about regular nursing round nurses working mornings evening night shifts form lectures questions answers group discussion surgical ward each session lasted minutes ensure appropriate implementation this intervention each nurses cared two patients based regular nursing round under supervision main researcher only supervised nurses main care provided educated nurses committed provide care patients regular nursing round.

In the intervention group, nurses from the second day until the end of fourth day of their hospitalization held regular nursing rounds every 2 h from 6 a.m. to 12 p.m. The division of patients in this ward was done based on the case method. The nurses in these rounds introduced themselves; appropriately communicated with the patients; assessed their needs and pain (by taking the face and location, and type of pain into account); made pain control interventions; provided defecation and urination, oral care, and medications; assessed vital signs; made available personal items; elevated bedside fences; answered any concerns and questions; used facilities such as appropriate mattresses and lotions; encouraged and helped them to move and change position to prevent ulcers; helped them to sit down and walk around; met their medical needs prescribed by the doctor and received orders; supported independence (encouraging family to visit the patient, encouraged participation in personal care, and helped the patient transfer); helped with bathing and keeping the patients clean and dry; and provided catheter, nutrition, and hydration care with recommended drinks. The nurses asked expression of patients' dissatisfaction and tried to make measures for patient's care and enhance their comfort, and examined their educational needs and met them before leaving the patients. In order to ensure the commitment of nurses to perform regular nursing rounds, this care program was supported by the nursing management of the hospital, and the head nurse supervised the strict implementation of this program. Also, a checklist was provided to the nurses and they were asked to complete it for each patient after the implementation of the program.

On the morning of fifth day of the patients’ admission in both groups, patients were completed PSNCQ and general comfort questionnaires. The workplace violence in the health sector questionnaire was completed by the nurses responsible for each patient care. To record the frequency of each threatening, verbal and physical violence by each patient and their relatives, the nurses were asked to use this questionnaire and at the end of each shift, they gave it to the nurses of next shift. This was continued from the second day to fourth day night (for three days) and finally the frequency of each violence was compared between the control and intervention groups.

### 2.6 Ethical considerations

This research was approved under the ethics code of IR.UMSHA.REC.1396.729 by the Ethics Committee affiliated with Hamadan University of Medical Sciences, Iran. The study was conducted in harmony with the ethical principles provided by the Statement of Helsinki and the guidelines of Iranian Ministry of Health and Medical Education. In this study, the selected participants were thoroughly informed about three objectives of the study and the study process. The subjects were informed about voluntariness, risk-free nature, confidentiality of their information, and the possibility of leaving the study at any time without affecting their care.

### 2.7 Data analysis

The collected data were analyzed using Kolmogorov Smirinov test, chi-square test, fisher's exact test, paired *t*-test, and independent *t*-test through SPSS version 16.0 (SPSS Inc., Chicago, IL, USA). A p-value less than 0.05 was considered as statistically significant.

## 3 Results

**Fig. 1:**
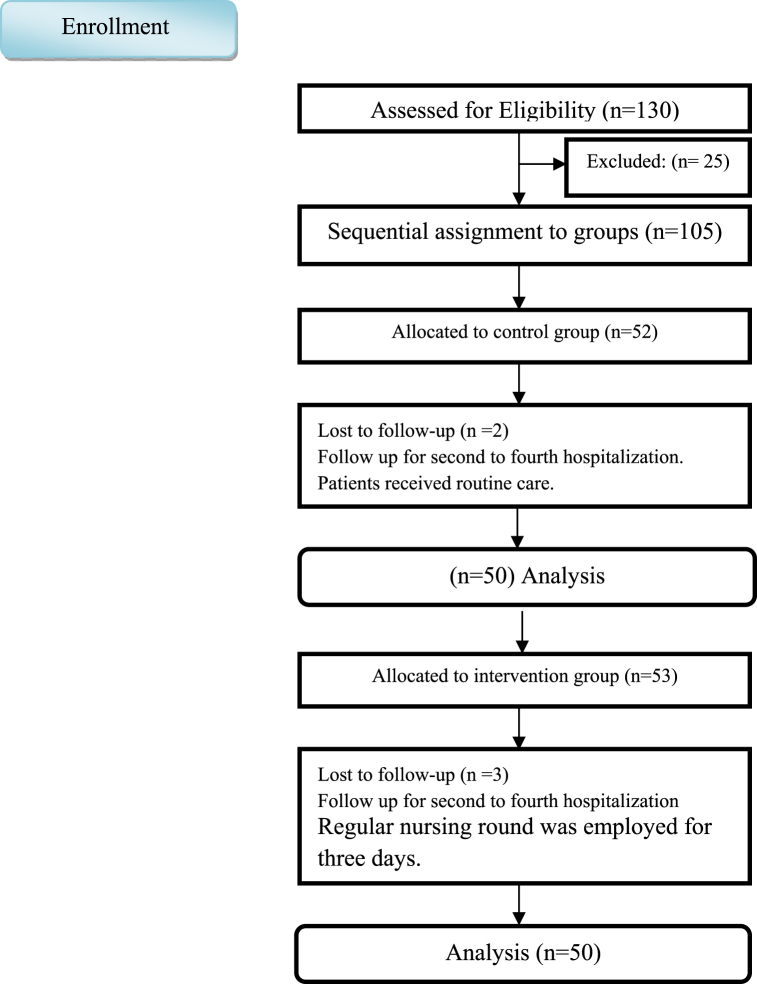
The study diagram.

A total of 105 patients were included in the study, 5 of whom were excluded from the study due to early discharge, and finally 50 patients completed the study in each group (see Fig. 1).

The chi-square test showed no statistically significant difference between the study groups in terms of their age, gender, marital status, educational level, having surgery, and history of hospitalization during the last 5 years. Therefore, the groups were homogeneous in terms of these variables (p > 0.05) (see Table 1).

**Table 1:** Comparison of demographic variables in the study groups.

Subjects	Characteristics	Categories	Intervention group (n = 50)	Control group (n = 50)	X^2^	P Value**
			n (%)	n (%)		
**Patients**	**Gender**	Female	26 (52)	25 (50)	0.04	1*
		Male	24 (48)	25 (50)		
	**Age**	18–30	14 (28)	14 (28)	0.80	0.847*
		31–50	20 (40)	23 (46)		
		51–70	7 (14)	7 (14)		
		>70	9 (18)	6 (12)		
	**Marital status**	Single	10 (20)	10 (20)	0.72	0.697**
		Married	36 (72)	38 (76)		
		Widowed/Divorced/Separated	4 (8)	2 (4)		
	**Educational status**	Illiterate	17 (34)	12 (24)	3.70	0.296*
		Primary	20 (40)	20 (40)		
		Diploma	6 (12)	9 (18)		
		Collegiate	7 (14)	9 (18)		
	**Job status**	Jobless	7 (14)	7 (14)	4.84	0.305*
		Housewife	19 (38)	17 (34)		
		Manual Worker	3 (6)	7 (14)		
		Employee	2 (4)	6 (12)		
		Free	19 (38)	13 (26)		
	**Economic status**	Bad	16 (32)	19 (38)	2.70	0.441**
		Medium	31 (62)	26 (52)		
		Good	3 (6)	3 (6)		
		Very Good	0 (0)	2 (4)		
	**History of hospitalization**	Yes	19 (38)	15 (30)	0.30	0.581*
		No	31 (62)	35 (70)		
	**Having surgery**	Yes	46 (92)	47 (94)	0.154	0.695**
		No	4 (8)	3 (6)		
**Nurses (n = 35)**	**Characteristics**	**Categories**	**Both group n (%)**			
	**Gender**	Female	24 (68.6)			
		Male	11 (31.4)			
	**Work experience**	1–6	18 (51.4)			
		7–12	10 (28.6)			
		13–18	5 (14.3)			
		>19	2 (5.7)			
	**Marital status**	Single	13 (37.1)			
		Married	20 (57.2)			
		Widowed/Divorced/Separated	2 (5.7)			
	**Educational status**	Bachelor	30 (85.7)			
		Master	5 (14.3)			

Note. *Chi-square test, **Fisher's exact test.

Table 2 shows the demographic characteristics of included nurses in the research, which were similar in both control and intervention groups (see Table 1).

**Table 2:** Comparison of the mean scores of satisfaction with nursing care and comfort in patients in the groups on the 2nd and 5th days.

Variables		Time of evaluation	groups		MD***	95% CI****		P value**
Main	Dimension		Intervention M ± SD	Control M ± SD		Lower	Upper	
**Satisfaction**	Knowledge & Professional Skills	Before	19.38 ± 3.34	20.14 ± 9.95	−0.76	−2.44	0.92	0.371
		After	26.96 ± 2.94	21.06 ± 4.53	5.90	4.38	7.42	>0.001
		P value*	0.001>	0.002				
	Communication	Before	11.56 ± 2.16	12.00 ± 3.45	−0.44	−1.58	0.70	0.448
		After	15.32 ± 1.80	12.72 ± 3.14	2.60	1.58	3.62	>0.001
		P value*	0.001>	0.116				
	Nursing Ethics	Before	14.12 ± 2.72	15.04 ± 3.95	−0.92	−2.27	0.42	0.178
		After	19.86 ± 1.53	16.02 ± 4.04	3.84	2.63	5.05	>0.001
		P value*	0.001>	0.052				
	Patient Education	Before	12.70 ± 3.01	13.88 ± 3.64	−1.18	−2.50	0.14	0.080
		After	20.02 ± 1.80	14.18 ± 4.03	5.84	4.60	7.08	>0.001
		P value*	>0.001	0.554				
	Total Satisfaction	Before	57.76 ± 8.64	61.06 ± 14.12	−3.30	−7.95	1.35	0.182
		After	82.16 ± 6.38	63.98 ± 12.97	18.18	14.122	22.24	>0.001
		P value*	>0.001	0.029>				
**General Comfort**	Physical comfort	Before	14.46 ± 2.03	14.98 ± 2.17	−0.52	−1.35	0.32	0.219
		After	16.52 ± 2.13	15.54 ± 2.25	0.98	0.11	1.85	>0.001
		P value*	0.001>	0.003				
	Environment comfort	Before	16.44 ± 2.61	15.76 ± 2.38	0.68	−0.31	1.67	0.177
		After	17.78 ± 2.56	16.20 ± 2.14	1.58	0.64	2.51	0.001
		P value*	0.001>	0.002				
	Psychological comfort	Before	16.90 ± 2.83	17.26 ± 3.10	−0.36	−1.53	0.89	0.546
		After	19.34 ± 3.79	17.80 ± 3.25	0.03	1.54	0.14	>0.032
		P value*	0.001>	0.002				
	Total general Comfort	Before	47.80 ± 4.45	48.00 ± 5.34	−0.20	−2.15	1.75	0.839
		After	53.64 ± 4.94	49.54 ± 5.42	4.10	2.041	6.16	0.001>
		P value*	0.001>	0.001>				

*Paired T Test, ** Independent T Test, ***Mean Difference, ****95% Confidence Interval of the Difference.

The investigators used the Kolmogorov-Smirnov test for checking the normality of the distribution of data. The results of the Kolmogorov-Smirnov test for the pre-test and post-test of control and experimental groups showed that p > 0.05, so the distribution of data is normal. So according to the normality of the variables, parametric tests were used.

The results of independent *t*-test showed that prior to the study, there was no statistically significant difference between the mean of satisfaction and the quality of nursing care and its dimensions between the control and intervention groups (p˃0.05). However, after the intervention, the mean of satisfaction and its dimensions in the intervention group was higher than the control group, which was statistically significant (p˂0.05). Additionally, the results of paired *t*-test showed that in the intervention group, the mean satisfaction and its dimensions after the intervention had a significant increase compared to before that (p˂0.05). However, in the control group, only the mean knowledge and professional skills dimension and total satisfaction had a significant increase (p˂0.05). The results of independent *t*-test showed that prior to the study, there was no statistically significant difference between the mean general comfort of the patients and its dimensions between the control and intervention groups (p˃0.05). But, after the intervention, the mean general comfort and its dimensions in the intervention group were higher than the control group, which was statistically significant (p˂0.05). Moreover, the results of paired *t*-test showed that in both control and intervention groups, the mean total comfort of patients and its dimensions significantly increased after the intervention compared to before that (p˂0.05) (See Table 2).

The Chi-square test showed statistically significant differences between the frequency of violence against nurses between the two study groups (p = 0.041), so that nurses in control group experienced more violence. There was no difference in type of violence between these two groups (p = 0.072) (See Table 3).

**Table 3:** Comparison of the frequency of violence and type of violence against nurses in two groups.

Characteristics	Categories	Intervention group	Control group	X^2^	*P Value
		n (%)	n (%)		
**Violence Against Nurses**	Yes	5 (9.3)	14 (28.0)	4.15	0.041
	No	45 (90.7)	36 (72.0)		
**Type Of Violence**	Verbal violence	4 (8)	11 (22)	5.26	0.072
	Threat	1 (1.3)	3 (6)		
	Physical	0 (0.0)	(0.0)0		
	Sexual harassment	0 (0.0)	(0.0)0		

Note. * Fisher's exact test.

## 4 Discussion

The results of this study show that implementation of regular nursing rounds for three days had a positive and significant effect on increasing satisfaction with nursing care quality and general comfort and decreasing violence rate against nurses in surgical ward in general. In agreement with our findings, Davis et al. [29] and Kessler et al. [30] have shown that regular nursing round increased patients’ satisfaction.

Mitchell et al. have found that regular nursing rounds reduced patients' falling down and use of nursing alarm, and increased patient's satisfaction [14]. However, differences were not statistically significant, which may possibly be related to fact that patient satisfaction was affected by treatment process, outcome, demographic characteristics, mental and physical statuses, and patient's expectations [31]. The results of this study were not consistent with those of study by Gardner et al. [18], which can be due to differences in number of samples, nature of disease, and used questionnaires. In addition, making significant changes to increase patients' satisfaction with low and moderate levels of satisfaction is difficult.

In control group, mean score of total satisfaction and all dimensions increased on fifth day compared to second day but only mean scores of knowledge and professional skills and total satisfaction were significant. Correspondingly this can be due poor past experiences of patients an overcrowded environment having critically-ill patients lack proper communication with patients Additionally some studies showed an inverse relationship between length stay hospital patient's satisfaction indicating relationship between long-term hospitalization patients' satisfaction score days near discharge lower than first day [32].

The patients' comfort in the two groups increased after the intervention period, which seems normal due to the passage of time from surgery and solving the main problem of patients and symptoms of their disease; however, in the intervention group, this increase was greater, which may possibly be due to the implementation of regular nursing round; paying more attention; and meeting the patients' physical, environmental, and psychological needs. Miu et al. have shown that pain relief for patients could increase the sense of patients' comfort [33]. In the current study, the patients' needs were met through timely pain relief and changing their positions. Gardner pointed out that the goal is to create a regular round for patients’ comfort, not the provision of complex care. Therefore, the results of this study show that this important goal was achieved [18].

If the patient feels supported by the nurse, he will feel more comfortable and healthy. The findings of the current research and a review of the studies show that the patients' perception of feeling comfortable, being taken care of, being a good nurse, etc., are concepts that, despite having slight differences among different cultures, have many points in common [8].

The nursing round significantly reduced violence against nurses in the intervention group, so that violence against nurses by the patients or their relatives in the intervention group decreased to one third compared to the control group, but no difference was reported in terms of the violence type. The most common type of violence against nurses was verbal violence and no physical violence was observed in this regard. The results of previous studies showed that nursing education could reduce violence against nurses [34,35]. Accordingly, in the study by Rahimi et al. communication skills training in the emergency department reduced violence against nurses, which was consistent with the results of this study [36]. One of the main causes of violence against nurses seems to be lack of care in emergencies, spending inadequate time for each patient, insufficient pain relief, and poor communication. These problems have been solved using regular nursing round program, and violence reduced as a result.

Advantages of this study over the studies by Negarandeh et al. [3] and Hutchings et al. [37] were the nurse's effective communication with the patients, careful assessment, effective interventions to meet their needs, and evaluation of the goals, which were performed every 2 h. On the other hand, meeting the patient's basic needs, having a person who was ready to respond to their problems and needs at any time, and relieving their mental and emotional pains increased their senses of satisfaction and comfort.

### 4.1 Study strengths and limitations

The strengths of this study include evaluating the impact of the nursing round on reducing nursing violence, the two-group nature of the study, its implementation by the nurses themselves, the easy-to-use regular nursing round by the nurses, the use of valid and objective questionnaires, and self-reports as a tool for comfort and satisfaction with care.

The most important limitations of this present study were non-random selection of subjects and time interval in sampling between control and intervention groups which were inevitable due intervention nature Moreover overcrowded ward might have affected intervention implementation In addition there need measure long-term results intervention As well this study done general surgical ward generalizability its results also subjected this limitation.

## 5 Conclusion

Regular nursing rounds can play important roles in improving patients' satisfaction with nursing care quality, patients’ comfort, and reducing violence against nurses. Therefore, nurse managers are recommended encourage educate design implement it clinical setting Nurse Managers should apprehend significance performing regular nursing round clinical setting Therefore it recommended nurse managers use findings current study order design implement evaluate regular nursing round improving nursing care.

## Author contribution statement

Zohre Roustaei, Narges Sadeghi, Azim Azizi, Mostafa Eghbalian, Sahar Dehdar Karsidani conceived and designed the experiments:

AA, ZR, ME; performed the experiments:

ZR, NS; analyzed and interpreted the data:

ZR, AA; contributed reagents, materials, analysis tools or data:

ME, SDK, AA; wrote the paper.

## Data availability statement

The data that has been used is confidential.

## Declaration of competing interest

The authors declare that they have no known competing financial interests or personal relationships that could have appeared to influence the work reported in this paper.
